# The Acute Effects of a Dopamine D3 Receptor Preferring Agonist on Motivation for Cigarettes in Dependent and Occasional Cigarette Smokers

**DOI:** 10.1093/ntr/ntx159

**Published:** 2017-07-13

**Authors:** Will Lawn, Tom P Freeman, Katie East, Annie Gaule, Elizabeth R Aston, Michael A P Bloomfield, Ravi K Das, Celia J A Morgan, H Valerie Curran

**Affiliations:** 1 Clinical Psychopharmacology unit, University College London, London, UK; 2 Addictions Department, Institute of Psychiatry, Psychology and Neuroscience, King’s College London, London, UK; 3 Psychiatric Imaging Group, Medical Research Council Clinical Sciences Centre, Hammersmith Hospital, London, UK; 4 Division of Psychiatry, University College London, Maple House, London, UK; 5 Department of Psychology, University of Exeter, Washington Singer Building, Exeter, UK; 6 Center for Alcohol and Addiction Studies, Brown University School of Public Health, Providence, RI

## Abstract

**Background:**

Dopaminergic functioning is thought to play critical roles in both motivation and addiction. There is preliminary evidence that dopamine agonists reduce the motivation for cigarettes in smokers. However, the effects of pramipexole, a dopamine D3 receptor preferring agonist, have not been investigated.

The aim of this study was to examine the effects of an acute dose of pramipexole on the motivation to earn cigarettes and nondrug rewards.

**Methods:**

Twenty dependent and 20 occasional smokers received 0.5 mg pramipexole using a double-blind, placebo-controlled crossover design. Motivation for cigarettes and consummatory nondrug rewards was measured using the DReaM-Choice task, in which participants earned, and later “consumed,” cigarettes, music, and chocolate. Demand for cigarettes was measured using the Cigarette Purchase Task (CPT). Self-reported craving, withdrawal, and drug effects were also recorded.

**Results:**

Dependent smokers chose (*p* < .001) and button-pressed for (*p* < .001) cigarettes more, and chose chocolate less (*p* < .001), than occasional smokers. Pramipexole did not affect the number of choices for or amount of button-pressing for any reward including cigarettes, which was supported by a Bayesian analysis. The dependent smokers had greater demand for cigarettes than occasional smokers across all CPT outcomes (*p*s < .021), apart from elasticity. Pramipexole did not affect demand for cigarettes, and this was supported by Bayesian analyses. Pramipexole produced greater subjective “feel drug” and “dislike drug” effects than placebo.

**Conclusions:**

Dependent and occasional cigarette smokers differed in their motivation for cigarettes but not for the nondrug rewards. Pramipexole did not acutely alter motivation for cigarettes. These findings question the role of dopamine D3 receptors in cigarette-seeking behavior in dependent and occasional smokers.

**Implications:**

This study adds to the growing literature about cigarette versus nondrug reward processing in nicotine dependence and the role of dopamine in cigarette-seeking behavior. Our results suggest nicotine dependence is associated with a hypersensitivity to cigarette rewards but not a hyposensitivity to nondrug rewards. Furthermore, our results question the importance of dopamine D3 receptors in motivational processing of cigarettes in occasional and dependent smokers.

## Introduction

Mesocorticolimbic dopamine functioning putatively plays a critical role in the reinforcing effects of recreational drugs, including nicotine,^1^ and the motivation for and learning about nondrug rewards.^2–4^ Over the past few decades, drug addiction has come to be conceptualized as a condition stemming from perturbations in the dopamine system.^5–7^ More recently, theories of addiction have emphasized the apparent concomitant increase in sensitivity to drug reward and decrease in sensitivity to nondrug reward.^8^ Dopamine is hypothesized to underpin this balance between drug and nondrug reward processing.^9^ Nicotine dependence has been associated with neuroadaptations in the dopamine system,^10,11^ a neural insensitivity to monetary reward^12,13^ and an imbalance in the processing of cigarette and nondrug rewards.^14–16^

Manipulation of the dopamine system is thus a promising avenue for the treatment of nicotine dependence. Indeed, bupropion, a dopamine and noradrenaline reuptake inhibitor, is efficacious in treating nicotine dependence. Bromocriptine, a dopamine D2 receptor preferring agonist, has been shown to acutely reduce ad libitum cigarette smoking,^17^ while extended use of bromocriptine was also associated with reduced cigarette smoking.^18^

Pramipexole is a non-ergot-derived dopamine agonist that binds to dopamine D2, D3, and D4 receptors, with the greatest affinity for the D3 receptor.^19^ Pramipexole is primarily used to treat Parkinson’s disease due to its activation of dopamine receptors in the degenerating basal ganglia. At low doses (e.g., 0.5 mg oral in humans), pramipexole is thought to act primarily on presynaptic autoreceptors such that it has an inhibitory effect on phasic dopamine firing.^20–22^ This reduction in phasic firing could theoretically lessen craving and hence decrease motivation for cigarettes.^23^ Indeed, in nicotine-dependent individuals, 0.5 mg of pramipexole reduced attentional bias to cigarette images^24^ while enhancing motivation for monetary reward.^25^

Aspects of both cigarette^26,27^ and nondrug reward processing^16,28,29^ significantly predict the success of smokers who are attempting to quit. Therefore, pharmacological treatments for nicotine dependence that simultaneously reduce motivation for cigarettes and augment motivation for alternative, nondrug rewards may be particularly efficacious.

In this study, we examined whether a single low (0.5 mg oral) dose of pramipexole would reduce dependent and occasional cigarette smokers’ motivation for cigarettes. We utilized a newly designed task, the Drug Reward and Motivation-Choice (DReaM-Choice) task,^14^ which measures motivation for both cigarette and consummatory nondrug rewards, using choices and repeated button-pressing. The DReaM-Choice has been shown to be sensitive to the level of nicotine dependence and acute nicotine abstinence. We also used a hypothetical cigarette purchase task (CPT),^30^ which is a behavioral economic task that asks participants how many cigarettes they would buy for increasing amounts of money. Behavioral economics views addiction as a state in which the substance acquires higher value than alternative rewards; the CPT measures the relationship between cigarette consumption and cost, that is, demand for cigarettes. Purchase tasks are analogous to progressive ratio self-administration tasks, but they are more efficient and have been used successfully in tobacco^30^ and marijuana^31^ users.

We hypothesized that:

Pramipexole would reduce motivation for cigarettes and increase motivation for nondrug rewards in the DReaM-Choice task.
Pramipexole would reduce demand for cigarettes in the CPT.
Compared to occasional smokers, dependent smokers would be more motivated for cigarettes on the DReaM-Choice task and exhibit greater demand for cigarettes in the CPT.
Pramipexole would have differential effects on the occasional and dependent smokers’ motivation to smoke cigarettes in both tasks. We did not hypothesize the direction of this interaction.

## Methods

### Design and Participants

A double-blind, placebo-controlled, crossover design with a between-subjects factor of group (dependent and occasional) and a within-subject factor of drug (placebo and pramipexole) was used. Twenty dependent (10 women) and 20 occasional (10 women) cigarette smokers took part in the study. A power analysis showed that a total sample size of 22 would be sufficient to detect a between-within interaction of medium effect size (*f* = 0.25) and a correlation between repeated measures of 0.7 (based on Lawn et al.^14^), with an α of 0.05 and a power of 0.8. However, we proceeded with the larger total sample size of 40 as used previously with the DReaM-Choice task,^14^ which gave us a power of 0.98 to detect an interaction with an effect size of f = 0.25. Having said that, if the correlation between repeated measures in this study was not as high as it was in the previous study (0.7), the power to detect the interaction would drop from 0.98.

Dependent smokers smoked on average ≥10 cigarettes/day and had a score of ≥5 on the Fagerstrom Test of Nicotine Dependence (FTND). Occasional smokers smoked on average 0.5–5 cigarettes per week and had an FTND score of 0. More detailed eligibility criteria can be found in the Supplementary material. The experiment was approved by University College London ethics committee, and all participants gave informed consent.

### Assessments

#### DReaM-Choice

**Figure 1.**
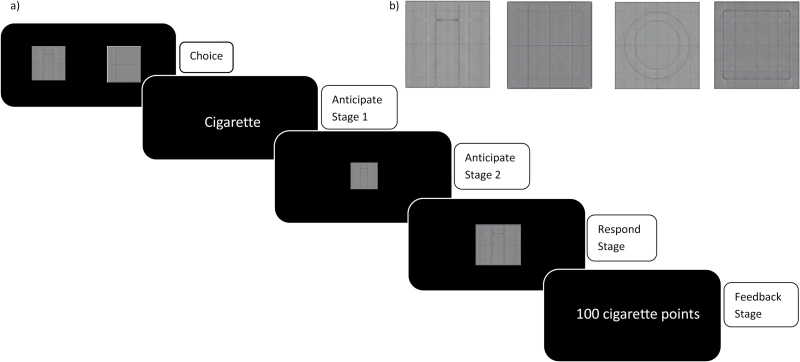
(A) Diagrammatic representation of a single trial of the DReaM-Choice Task. It was designed to determine (1) the number of choices for each reward, which indexes “relative preference” and (2) the average number of button-presses for each reward, which indexes motivation. During the “choice stage”, the cues were presented and a choice was made with button F (left option) or J (right option; unlimited time); during the “anticipate stage 1”, the word of the reward, for example, “cigarette,” was shown (0.5s); during the “anticipate stage 2,” a small version of the cue was shown (4s); during the “respond stage,” the spacebar was pressed as many times as desired with the nondominant little finger in 7s,^32^ in order to win points for the chosen reward; during the “feedback stage,” feedback concerning the amount of points won was provided for 1s. Each of the 6 possible choices were presented 4 times in three blocks, making a total of 72 trials, with trial order pseudo-randomized and left/right cue position counterbalanced. (B) The cues used in the DReaM-Choice task to represent each reward. From left to right: cigarette, chocolate, music, and paper. The “delivered rewards” which were actually given to participants to consume after the DReaM-Choice task finished were Malboro Gold cigarettes (tar 6 mg and nicotine 0.5 mg), Cadbury’s Dairy Milk chocolate, individually chosen music,^33^ and pieces (~2cm^2^) of lined paper. Paper was included as a control commodity to demonstrate that the rewards were motivating relative to a control commodity.^14,34^

The DReaM-Choice (Figure 1)^14^ task involves participants making a series of two-option choices between different rewards (cigarette, music, chocolate, and paper—a neutral commodity) and then working for the chosen option via repeated button-pressing. Points for each reward are accumulated throughout the task and are exchanged for *real* delivered rewards after the task has been completed, which can then be consumed in the laboratory.^14^ The Supplementary materials provide a detailed description of the updated version of the task.

In brief, the DReaM-Choice task measures two main outcomes: (1) the number of choices for each reward and (2) the average number of button-presses for each reward. Figure 1 depicts an example trial in which there is a choice between cigarette and chocolate, the cigarette is chosen, and then subsequently worked for.

After the task, participants received their delivered rewards and had 20 minutes to consume them. Every time they consumed 1 unit (¼ cigarette, 30s music, ½ chunk of chocolate) of a reward, they reported their subjective liking (rated from −10 “extremely dislike” to +10 “extremely like”). However, we report only their subjective liking of the first “unit” of each reward consumed due to satiation effects.

#### Cigarette Purchase Task

The CPT^30^ assesses cigarette demand, or in other words, the relationship between cigarette consumption and cost.^30^ It is an analogue of a progressive ratio operant task, as consumption is investigated under progressively increasing financial cost. It is an established and well-validated task that is used to examine the behavioral economic concept of “demand” relating to cigarettes.^30,35^ In this version, participants were asked how many cigarettes they would hypothetically buy for the next 3 hours at increasing prices.^36^ Participants were asked “How many cigarettes would you smoke if they were _____ each”. Prices included: £0 (free), 1p, 2p, 5p, 10p, 15p, 20p, 25p, 30p, 35p, 40p, 45p, 50p, 60p, 70p, 80p, 90p, £1, £2, £3, £4, and £5 and were presented in that order. The CPT generates five indices of cigarette demand: breakpoint (cost suppressing consumption to zero), intensity (amount of drug consumed at zero cost), O_max_ (peak expenditure), P_max_ (price at maximum expenditure), and elasticity (the slope of the demand curve); see Supplementary material for more details.

## Self-Rated Assessments

### State Measures

#### *Tobacco Craving Questionnaire-Short Form (TCQ-SF*)

This craving scale^37^ consists of 12 items that are rated “right now” from 1 (strongly disagree) to 7 (strongly agree). There are four subscales: emotionality, expectancy, compulsivity, and purposefulness. Higher scores reflect greater tobacco craving.

#### *Mood and physical symptoms scale (MPSS*)

This scale^38^ consists of 7 items that assess nicotine withdrawal symptoms. Five items were rated “right now” from 1 (not at all) to 5 (extremely): “depression,” “irritability,” “restlessness,” “hunger,” and “poor concentration.” Two items were rated “in the past two hours”: “how much have you felt the urge to smoke?” from 0 (not at all) to 5 (all the time) and “how strong have these urges been?” from 0 (no urges) to 5 (extremely strong). Higher scores reflect greater withdrawal symptom severity.

#### *Drug Effects Questionnaire (DEQ*)

This assessment^39^ comprised 5 visual analogue scales (VAS) rated according to how the participant feels “right now” from 0 mm (“not at all”) to 100 mm (“extremely”): (1) “do you feel a drug effect”; (2) “are you high”; (3) “do you dislike any of the effects”; (4) “do you like any of the effects,” and (5) “would you like more”.

#### Nausea and Drowsiness

Participants reported how nauseous and how drowsy they were from 0 “not at all” to 10 “extremely.”

#### Time Since Last Smoked

Participants were asked whether they had smoked tobacco on the day of testing and the day before testing. If the answer to either of these questions was yes, they stated how long ago they last smoked tobacco.

### Trait Measures

These measures assessed depression, tobacco/nicotine dependence, history of drug use, and anhedonia. A full description of the trait measures used can be found in the Supplementary material.

### Procedure

Participants were screened on the telephone to determine if they met the eligibility criteria. Eligible participants attended two 3.5-hour sessions separated by a washout period lasting between 7 and 25 days (mean = 9, SD = 4.4). Participants were told to smoke “normally” before the experiment; they were allowed to smoke (or not) before they arrived, depending on what was normal for them. Participants were also asked to fast for an hour beforehand and to avoid driving or operating heavy machinery on the day of testing. First, participants provided a carbon monoxide (CO) reading and completed the state questionnaires (excluding the DEQ). Pramipexole (0.5mg; peak plasma levels 1-3 h)^40^ or matched placebo (lactose powder) was then orally administered. Based on previous research,^25,41^ 30 mg of the peripheral dopamine D2 antagonist domperidone was orally administered on both sessions to reduce unwanted side effects such as nausea. Previous research suggests minimal central effects of domperidone,^42^ as it cannot cross the blood–brain barrier.^43^ Therefore, we assumed domperidone would not affect reward processing, and previous researchers have made this assumption too.^24,25,41^ The nausea experienced by the participants had domperidone not been administered would have made the experiment unfeasible. Immediately after drug administration, participants completed half of the trait questionnaires, which were split across the two sessions. Testing began 90 minutes postdrug administration. Assessments were conducted in the following order: state questionnaires (90 minutes), CPT (100 minutes), other tasks that will be described elsewhere (105 minutes), DReaM-Choice task (145 minutes), reward consumption (175 minutes), and state questionnaires (195 minutes). Given the participants could have smoked just before taking part in the experiment, the minimum length of nicotine abstinence before the CPT was therefore approximately 1 hour 40 minutes, and the minimum length of nicotine abstinence before the DReaM-Choice task was approximately 2 hours 25 minutes. Smoking was not permitted until the consumption stage of the experiment. Participants were reimbursed £7.50/h.

### Statistical Analyses

All data were analyzed using IBM Statistical Package for Social Sciences (IBM SPSS version 22) and Graphpad Prism 6, for the Cigarette Purchase Task data.

The majority of data were analyzed using the general linear model. Where residuals were not normally distributed or the group variances were not homogenous, nonparametric tests were used when available and appropriate. In repeated-measures analysis of variance [ANOVA], when sphericity was violated, the Greenhouse-Geisser correction was used, and corrected degrees of freedom are reported. In order to explore significant interactions, a Bonferonni correction was applied to post hoc comparisons via the syntax in SPSS. Drug order was included in all of the ANOVAs to determine if its presence affected the pattern of results. It did not, so the results are reported without drug order included. Given Beck Depression Inventory (BDI) scores differed between groups, we included this as a covariate after each analysis. In order to evaluate evidence in favor of null hypotheses, scaled Jeffreys-Zellner-Siow (JZS) Bayes factors were calculated using an online calculator (http://pcl.missouri.edu/bayesfactor). We used a scaled-information prior of *r* = 1, which is the default value recommended.^44^

A detailed description of the statistical analyses used for each set of data can be found in Supplementary material.

## Results

### Demographics

The dependent smokers and occasional smokers had similar average ages of 24.4 (SD = 6.8) and 22.6 (SD = 3.79), respectively. The dependent smokers had greater dependence than the occasional smokers on the FTND (5.7 vs. 0, U_38_ = 0.00, *p* < .001) and the Cigarette Dependence Scale (CDS; 18.5 vs. 7.7, t_37_=11.923, *p* < .001). The dependent smokers smoked more cigarettes per day and week (16.5 vs. 0.5, U_38_ = 0.00, *p* < .001), started smoking at an earlier age (13.4 vs. 15.0, t_38_ = 2.504, *p* = .017) and reported greater subjective liking, in general, of smoking a cigarette (8.2 vs. 5.2, t_37_ = 4.687, *p* < .001; Supplementary Tables A and B ) compared with the occasional smokers. There was no evidence that the groups differed on any nonsmoking demographic variables apart from BDI, on which dependent smokers (10.80, SD = 7.50) had a greater score than occasional smokers (6.26, SD = 4.58; t_37_=2.265, *p* = .029.

### State Measures

Relative to placebo, pramipexole increased feelings of “feel drug” and “dislike drug” and decreased feelings of “want more drug.” See Supplementary material for the full drug effects questionnaire results.

#### TCQ

On each subscale and the total TCQ score (Table 1), there was an interaction between group and time, and main effects of both group and time. Dependent smokers, compared with occasional smokers, had greater craving on each subscale. Craving scores increased from predrug to postdrug and decreased from postdrug to postconsumption in dependent smokers, whereas craving scores stayed similar between predrug and postdrug and decreased from post-drug to post-consumption in occasional smokers. There was a main effect of drug on the compulsivity and purposefulness subscales, with greater scores on the pramipexole session than the placebo session.

**Table 1.** Group means (SD) for TCQ-SF predrug, postdrug, and postconsumption for placebo and pramipexole sessions for dependent and occasional smokers

		Dependent		Occasional		Group × Drug × Time	Group× Drug	Group × Time	Drug × Time	Group	Drug	Time
		Placebo	Pramipexole	Placebo	Pramipexole	*F*_2, 76_	*F*_1, 38_	*F*_2, 76_	*F*_2, 76_	*F*_1, 38_	*F*_1, 38_	*F*_2, 76_
TCQ emotionality	Predrug	8.40 (3.79)	9.30 (4.99)	5.90 (2.81)	5.15 (2.68)	0.904	1.116	13.683***	1.463	10.752***	1.532	20.989***
	Postdrug	9.25 (4.38)	10.80 (4.65)	5.10 (3.02)	5.90 (3.31)							
	Postconsumption	6.20 (3.19)	5.65 (2.85)	5.05 (3.41)	5.15 (2.81)							
TCQ expectancy	Predrug	13.25 (4.13)	14.65 (3.22)	10.10 (4.14)	9.75 (4.33)	0.286	3.035	14.170***	0.353	28.847***	0.217	49.825***
	Postdrug	16.40 (3.97)	16.85 (3.15)	9.70 (4.24)	8.75 (4.23)							
	Postconsumption	9.50 (3.91)	9.90 (3.26)	7.20 (3.69)	7.20 (2.98)							
TCQ compulsivity	Predrug	9.05 (4.19)	10.25 (4.32)	4.30 (1.98)	4.25 (1.83)	2.782	1.638	17.425***	0.979	31.812***	4.912*	23.998***
	Postdrug	9.90 (4.93)	12.35 (5.24)	4.55 (2.48)	4.55 (2.37)							
	Postconsumption	7.50 (4.19)	6.65 (3.79)	3.80 (1.79)	4.60 (2.60)							
TCQ purposefulness	Predrug	12.65 (2.89)	14.10 (3.19)	8.45 (2.84)	8.30 (3.31)	1.790	0.806	8.157 ***	0.427	48.088***	5.349*	36.362***
	Postdrug	13.30 (4.78)	15.35 (3.86)	7.35 (3.07)	7.70 (2.72)							
	Postconsumption	9.80 (4.62)	9.25 (3.81)	5.55 (2.66)	6.65 (3.00)							
TCQ total	Predrug	43.35 (12.88)	48.30 (13.02)	28.75 (8.97)	27.45 (9.53)	1.339	2.020	18.780***	0.263	37.186***	2.891	52.705***
	Postdrug	48.85 (15.19)	53.85 (15.79)	26.85 (10.36)	26.90 (9.66)							
	Postconsumption	33.00 (14.60)	31.45 (10.95)	21.60 (10.18)	23.60 (9.70)							

Abbreviation: TCQ, Tobacco Craving Questionnaire.

*p < .05; ***p < .001.

#### MPSS

“Depressed” scores decreased as the experiment progressed while “hungry,” “poor concentration” and “time spent with urges” increased as the experiment progressed (Supplementary Table C). The dependent smokers, compared with the occasional smokers, reported greater “time spent with urges” and “strength of urges to smoke.” There was a main effect of drug on “strength of urges to smoke,” with greater scores on the pramipexole session than the placebo session.

#### Nausea and Drowsiness

There were Drug × Time interactions for both nausea (*F*_2, 76_ = 5.863, *p* = .004, η^2^_p_ = 0.134) and drowsiness (*F*_2, 76_ = 9.699, *p* < .001, η^2^_p_ = 0.203). Exploration of the interactions showed that at postconsumption, for nausea, pramipexole led to higher ratings than placebo (t_38_ = 2.571, *p* = .014, mean difference = 0.900) and at postconsumption, for drowsiness, pramipexole led to higher ratings than placebo (t_38_=4.695, *p* < .001, mean difference = 2.000).

#### Time Since Last Smoked

On the placebo condition, all dependent smokers had smoked either on the day of testing (n = 18) or the day before (n = 2), and the mean time since last cigarette (relative to the start of the experiment) was 87.90 (SD = 217.94) minutes. On the placebo condition, six of the occasional smokers had smoked either on the day of testing (n = 3) or the day before (n = 3). Of those six, the mean time since last cigarette was 501.83 (SD = 556.35) minutes.

On the pramipexole condition, all dependent smokers had smoked on the day of testing, and the mean time since last cigarette was 29.30 (SD = 37.07) minutes. On the pramipexole condition, seven of the occasional smokers had smoked either on the day of testing (n = 1) or the day before (n = 6). Of those seven, the mean time since last cigarette was 915.05 (SD = 258.33) minutes.

The DReaM-Choice task was completed approximately 2 hours 25 minutes after the start of the experiment, so each participant’s nicotine abstinence was equal to 2 hours 25 minutes + their time since last smoked.

Neither group significantly differed in their time since last cigarette between placebo and pramipexole conditions. However, there was a main effect of group such that the dependent smokers had smoked more recently than the occasional smokers (*F*_1, 24_ = 55.972, *p* < .001, η^2^_p_ = .700).

### DReaM-Choice task

#### Choices

**Figure 2.**
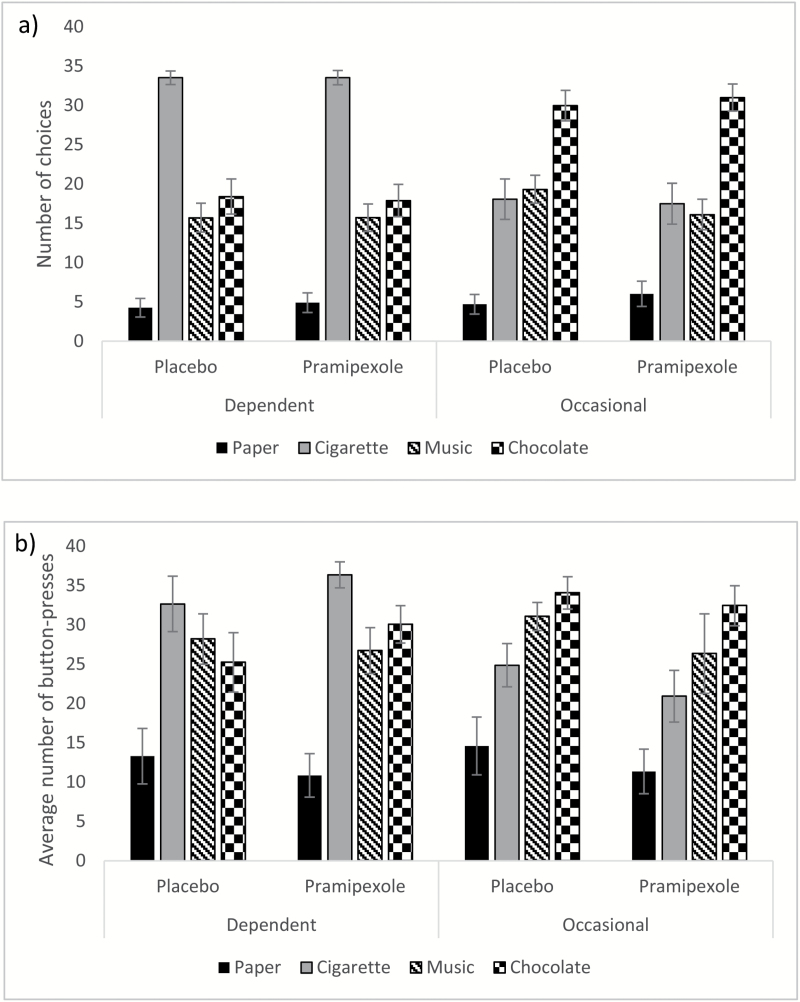
Group means for (A) The number of choices for each reward type and (B) the average number of button-presses for each reward type, in the DReaM-Choice, for dependent and occasional smokers on the placebo and pramipexole sessions. Error bars show standard error.

One occasional smoker’s data was missing due to a computer error (Figure 2a). A three-way ANOVA with a between-subjects factor of group and within-subjects factors of drug and reward was conducted. There was an interaction between group and reward (*F*_2.430, 89.927_ = 21.009, *p* < .001, η^2^_p_ = .362) and a main effect of reward (*F*_2.430, 89.927_ = 55.883, *p* < .001, η^2^_p_ = .602). Exploration of the Group × Reward interaction showed that dependent smokers chose cigarettes more (t_37_ = 7.259, *p* < .001, mean difference=15.737, 95% confidence interval [CI]: 11.344 to 20.129) and chocolate less (t_37_=4.702, *p* < .001, mean difference = 12.366, 95% CI: 7.038 to 17.694) than occasional smokers.

There was no three-way interaction (*F*_2.412, 89.251_ = 0.502, *p* = .660, η^2^_p_ = .013) and no interaction between drug and reward (F_2.412, 89.251_ = 0.550, *p* = .612, η^2^_p_ = .015) and no main effect of drug (*F*_1, 37_= .769, *p* = .386, η^2^_p_=0.020). Covarying for BDI had no effect.

For the effect pramipexole had on cigarette choices, within the dependent smokers, the Bayesian analysis showed that the null hypothesis was about 6 times more likely than the alternative hypothesis (JZS Bayes factor = 5.86), providing evidence that pramipexole did not affect their number of cigarette choices. Within the occasional smokers, the Bayesian analysis showed that the null hypothesis was about 6 times more likely than the alternative hypothesis (JZS Bayes factor = 5.63), providing evidence that pramipexole did not affect their number of cigarette choices.

#### Average Number of Button-Presses

One occasional smoker’s data was missing due to a computer error (Figure 2b). There were no differences between the groups or sessions in baseline button-pressing speed, so analysis continued without including this as a covariate. A three-way ANOVA with a between-subjects factor of group and within-subjects factors of drug and reward was conducted. There was an interaction between group and reward (*F*_3, 111_ = 6.999, *p* < .001, η^2^_p_ = .159) and a main effect of reward (*F*_3, 111_ = 35.373, *p* < .001, η^2^_p_ = 0.489). Exploration of the Group × Reward interaction showed that the dependent smokers pressed for cigarettes more (t_37_=3.663, *p* < .001, mean difference=11.613, 95% CI: 5.189 to 18.036) than the occasional smokers. There was no three-way interaction (*F*_3, 111_=35.373, *p* < .001, η^2^_p_=0.489), no interaction between drug and reward (*F*_2.229, 82.461_ = .976, *p* = .389, η^2^_p_=0.026), and no main effect of drug (*F*_1, 37_ = .348, *p* = .559, η^2^_p_=0.009). Covarying for BDI had no effect.

For the effect pramipexole had on button-pressing for cigarettes, within the dependent smokers, the Bayesian analysis showed that the null hypothesis was about 4 times more likely than the alternative hypothesis (JZS Bayes factor = 3.58), providing evidence that pramipexole did not affect button-pressing for cigarettes. Within the occasional smokers, the Bayesian analysis showed that the null hypothesis was about 3 times more likely than the alternative hypothesis (JZS Bayes factor = 3.36), providing evidence that pramipexole did not affect button-pressing for cigarettes.

See Supplementary material for the “time taken to choose a reward” results.

#### Liking of First Reward Unit Consumed

There was an interaction between group and reward (*F*_2, 130.476_ = 4.457, *p* = .013, η^2^_p_=0.064) and a main effect of group (*F*_1, 41.759_ = 6.086, *p* = .018, η^2^_p_ = 0.226), with overall liking higher in the dependent smokers than the occasional smokers (Supplementary Figure A). The Group × Reward interaction was explored by conducting mixed effects models within each reward separately. Dependent smokers liked cigarettes more than occasional smokers (*F*_1, 26.073_ = 19.738, *p* < .001, η^2^_p_=0.431), but there were no differences for music (*F*_1, 28.799_=1.567, *p* = .221, η^2^_p_=0.0516) or chocolate (*F*_1, 32.330_ = 0.140, *p* = .710, η^2^_p_=0.004). There was a trend main effect of drug with liking ratings marginally higher following pramipexole compared with placebo (*F*_1, 122.249_ = 3.175, *p* = .077, η^2^_p_ = 0.0253) but no Drug × Reward interaction (*F*_2, 121.737_ = 0.388, *p* = .679, η^2^_p_ = .006).

### CPT

**Figure 3.**
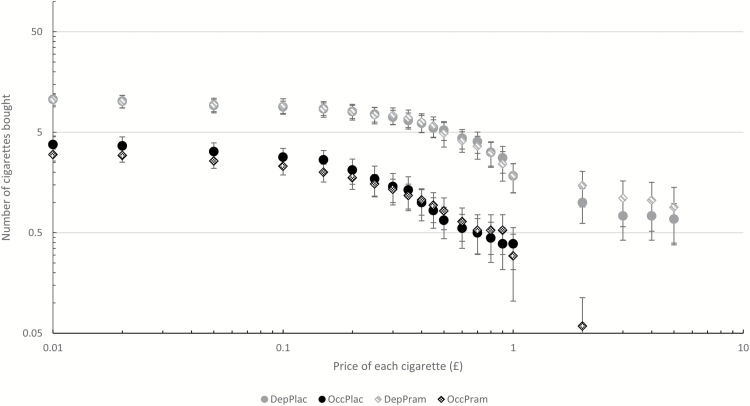
Cigarette demand curve from the Cigarette Purchase Task (CPT) for dependent and occasional smokers on the placebo and pramipexole sessions. Error bars show standard error. DepPlac = Dependent smokers on placebo; OccPlac = Occasional smokers on placebo; DepPram = Dependent smokers on pramipexole; OccPram = Occasional smokers on pramipexole.

One dependent smoker and three occasional smokers were excluded for stating they would buy the same amount of cigarettes for every price (Figure 3). Elasticity for an additional two occasional smokers could not be calculated by GraphPad Prism 6 due to an unsuitable range of data points. Breakpoint, intensity, O_max_, and P_max_ were all log_10_ transformed to improve the normality of their distributions and their residuals’ distributions. Correlations between the indices of demand can be seen in Table E of the Supplementary material. There were frequently high associations between them, as has been found in previous research.

A two-way ANOVA with a between-subjects factor of group and a within-subjects factor of drug was conducted for each CPT outcome variable. There was a main effect of group for breakpoint (*F*_1, 32_=21.764, *p* < .001, η^2^_p_=0.404), intensity (*F*_1, 32_=35.367, *p* < .001, η^2^_p_=0.525), O_max_ (*F*_1, 31_=25.147, *p* < .001, η^2^_p_=0.448), and P_max_ (*F*_1, 31_=18.740, *p* < .001, η^2^_p_=0.377). For all of these outcomes, dependent smokers had greater values than occasional smokers. There was no main effect of drug and no interaction between drug and group on any CPT metric (see Supplementary Table D).

Adding BDI as a covariate into the models showed that BDI did not interact with the drug effects; however, higher BDI levels were associated with greater demand for cigarettes (see Supplementary material).

For the effect of pramipexole on CPT metrics, within dependent smokers, the Bayesian analyses showed that the null hypotheses were more likely than the alternative hypotheses for breakpoint, intensity, O_max_, P_max_, and elasticity with JZS Bayes factors of 5.56, 2.14, 4.69, 1.08, and 2.39, respectively. Within the occasional smokers, the Bayesian analyses showed that null hypotheses were more likely than the alternative hypotheses for breakpoint, intensity, O_max_, P_max_, and elasticity with JZS Bayes factors of 5.26, 4.62, 2.98, 4.24, and 1.58.

### Correlations

Within the occasional smokers, subjective ratings of liking cigarettes in general predicted behavior on the DReaM-Choice task. However, this was not the case in the dependent smokers. See Supplementary material for full results.

### Fidelity of the Blind

The distribution of drug guesses (whether the participant was on placebo or pramipexole) was marginally different from that of chance as determined by the McNemar test (*p* = .052), suggesting there was some evidence that participants could tell what drug they had been given.

## Discussion

To the authors’ knowledge, this is the first study to examine whether pramipexole acutely influences motivation for real cigarettes. In dependent and occasional cigarette smokers, a single oral 0.5 mg dose of pramipexole did not affect the relative preference for, motivation for and liking of cigarettes in the DReaM-Choice task. Moreover, there was no evidence that pramipexole affected demand for cigarettes in the CPT or self-reported craving or withdrawal symptoms. Nor was there evidence that pramipexole influenced responding for the consummatory nondrug rewards in the DReaM-Choice task. Bayesian analyses, conducted within each group separately, showed there was evidence in favor of the null hypotheses that pramipexole did not affect motivation for cigarettes or cigarette demand in the CPT. Furthermore, pramipexole did not have different effects on occasional and dependent smokers’ motivation for cigarettes. In support of previous research,^14^ dependent smokers exhibited greater motivation for cigarettes than occasional smokers, while there was no evidence that dependent smokers were less motivated to receive chocolate or music.

### Pramipexole

In terms of pramipexole affecting choices, button-pressing, and demand for cigarettes, we found nonsignificant results, mostly small effect sizes and evidence from Bayesian analyses in favor of the null hypotheses. Our findings therefore question the role of the D2-subfamily of dopamine receptors, especially the D3 receptor in the motivation to smoke cigarettes. Given bromocriptine reduced ad libitum smoking in humans,^17^ D2-preferring agonists may be superior to D3-preferring agonists (pramipexole) in disrupting cigarette processing. However, their results could be partially due to large increases in nausea, rather than central dopamine receptor agonism. If nausea simply reduces cigarette smoking, the coadministration of domperidone may have dampened the effects of pramipexole. Interestingly, although we coadministered domperidone, the pramipexole condition did lead to higher ratings of nausea and drowsiness at postconsumption, so domperidone did not fully eradicate the emetic effects of pramipexole.

More generally, the focus on dopamine in addiction, particularly nonstimulant addiction, may be overstated.^5^ For instance, acutely administered nicotine has sometimes failed to provoke dopamine release in the striatum,^45,46^ and an unchanged striatal D2/D3 receptor density in cigarette smokers has been reported.^47^ Despite some positive findings concerning bromocriptine, other dopamine agonists and antagonists have failed to show effects on craving for cigarettes.^23^ Our null results lend support to the general hypothesis that nicotine addiction is more complex, in a neurobiological sense, than simply a dysfunctioning dopamine system.

Alternatively, it may be that chronic, rather than acute, administration of pramipexole is needed in order to more substantially manipulate the dopamine system such that motivation to smoke is lowered. Indeed, bupropion, an approved drug for aiding smoking cessation can increase smoking when given acutely^48^ but reduces smoking when given chronically.^49^ Importantly, pramipexole may have different effects in cigarette smokers who are motivated to quit from those who are not, like bupropion and nicotine replacement therapy.^50^

Previously, pramipexole (at this dose) has been shown to reduce the strength of urges to smoke.^24^ In contrast, craving in this study appeared to be greater during the pramipexole session compared with the placebo session (on two subscales of the TCQ-SF and the “strength of urges” subscale of the MPSS). However, there were no Drug ×Time interactions, and these effects appeared to be driven by seemingly random baseline differences (see Table 1 and Supplementary Table C). Hence, our results suggest that pramipexole neither reduced nor enhanced craving for cigarettes.

Pramipexole did not affect relative preference for, motivation for or liking of the consummatory nondrug rewards, music, and chocolate. Given its promotivational acute,^25^ we had hoped pramipexole would enhance nondrug reward processing, while impairing cigarette reward processing.^24,25^ This differential profile of drug and nondrug reward processing effects may have the most therapeutic benefits.^16,28^ However, not only did pramipexole fail to significantly alter processing of cigarette reward, it also failed to alter reward processing of the consummatory nondrug rewards.

### Group differences

In terms of group differences, the dependent smokers demonstrated hypersensitivity to cigarettes compared with the occasional smokers. The dependent smokers chose cigarettes more frequently, worked harder for them during the button-pressing stage and reported greater subjective liking when they consumed cigarettes. This replicates a previous comparison between these groups on the DReaM-Choice task.^14^ Despite augmented motivation for drugs being a hallmark of addiction, previous laboratory-based tests of cigarette self-administration have sometimes failed to differentiate addicted and nonaddicted smokers.^15,51^ Therefore, these data from the DReaM-Choice reveal that enhanced motivated responding for cigarettes in dependent versus occasional smokers can be reliably detected in the laboratory. Moreover, as would be expected, the forced period of nicotine abstinence during the experiment enhanced craving only in the dependent smokers, but not the occasional smokers, who would be used to periods much longer than this without smoking.

There was no evidence that dependent smokers were less motivated for chocolate or music than occasional smokers, as measured by button-pressing. This corroborates previous research^14,52^ that nicotine-dependent smokers do not appear to be amotivated for nondrug rewards following ad libitum smoking or short (approximately 2 hours 25 minutes in this study) nicotine abstinence. Whether or not more substantial nicotine abstinence (12-24 hours) produces nondrug reward processing deficits remains contentious.^14,15,52,53^

### Strengths and limitations

Key strengths of this study include the placebo-controlled, double-blind, crossover design; the relatively large sample size (n = 40), compared with other acute pramipexole studies^20,24^; the well-matched groups; the range of metrics we used to assess motivation to smoke cigarettes; and the comparison of real cigarettes with real nondrug rewards in the DReaM-Choice task. Another strength is that results from the DReaM-Choice task and the CPT dovetailed to demonstrate that pramipexole had no significant effect on motivation to smoke cigarettes. Moreover, the associations between performance on the DReaM-Choice task and the CPT demonstrate the DReaM-Choice task’s validity. A methodological improvement would have been the measurement of biological variables, such as pramipexole plasma levels^40^ so that the ability of the drug to enter the blood could have been verified. Future research should also investigate the effects of different doses of pramipexole on motivational processing.

### Summary

In conclusion, we found no evidence to suggest that an acute, low dose of pramipexole reduces motivation for cigarettes or redresses the imbalance of cigarette and non-drug reward processing in dependent cigarette smokers. Following a short period of nicotine abstinence (2 hours 25 minutes), dependent smokers appeared to be more motivated for cigarettes but no less motivated for consummatory nondrug rewards than occasional smokers. These findings may question the role of the D2-subfamily of dopamine receptors, especially the D3 receptor, in cigarette-seeking behavior in both dependent and occasional smokers; however, further pharmacological and imaging research is needed to fully understand what roles these receptors play in nicotine dependence.

## Supplementary Material

Supplementary data are available at *Nicotine and Tobacco Research* online.

## Funding

This study was funded by BBSRC PhD funding.

## Declaration of Interests

CJAM has consulted for Janssen and GlaxoSmithKline and received compensation. No other authors have anything to declare.

## Supplementary Material

Supplementary_Materials
