# Supplementary material for: The Acute Effects of a Dopamine D3 Receptor Preferring Agonist on Motivation for Cigarettes in Dependent and Occasional Cigarette Smokers
Source: Nicotine Tob Res. 2017 Jul 13;20(7):800–9. doi: 10.1093/ntr/ntx159 (PMC5991206; doi:10.1093/ntr/ntx159)
Supplement: Supplementary_Materials [file ntx159_suppl_supplementary_materials.docx]

**Title: The acute effects of a dopamine D3 receptor preferring agonist on motivation for cigarettes in dependent and occasional cigarette smokers**

**Supplementary materials**

***Methods***

**Eligibility criteria**

Inclusion criteria were: (1) smoke on average ≥10 cigarettes/day (for dependent smokers) or smoke 0.5-5 cigarettes per week (for occasional smokers); (2) have an FTND score ≥5 (for dependent smokers) or 0 (for occasional smokers); (3) aged 18-50. Exclusion criteria were: (1) have been a regular, daily cigarette smoker in the past (for occasional smokers); (2) seeking treatment for a mental health problem; (3) using psychiatric medication; (4) use of an illicit drug once per week or more; (5) using a pharmacotherapy to quit smoking; (6) have a body mass index outside the range 18-30; (7) tumours of the adrenal or pituitary glands; (8) reduced functioning of the kidney or liver; (9) pregnant or breast-feeding; (10) hypersensitivity to pramipexole or domperidone; (11) current diagnosis of alcohol dependence; (12) not allergic to lactose; (13) be a vegan (due to the lactose used in placebo); (14) normal or corrected-to-normal vision.

**DReaM-Choice Task**

The DReaM-Choice task was programmed using Experiment Builder (SR Research, Ontario, Canada). It was designed to determine: (1) the number of choices for each reward, which indexes ‘relative preference’ and (2) the average number of button-presses for each reward, which indexes motivation.

During each choice, two cues, which each represent one type of reward, are presented side by side (see figure 2 for the cues). On each trial, participants: make a choice between two reward types (unlimited time), see the word of the selected reward type (0.5s), anticipate working for the reward (4s), work for the reward by pressing the spacebar with the non-dominant little finger (7s) and receive feedback about how many points were won (1s). The more times they press the spacebar in 7s, the more points that are earned for the chosen reward. There were 72 trials in total, split into 3 separate blocks.

Before participants started the actual task, we measured how many times they could press the spacebar with their non-dominant little finger in 7s. This was done three times and an average was taken. This allowed us to: (1) check that pramipexole or group didn’t affect the speed at which they could press a button and (2) weight the number of points won according to their baseline button-pressing speed.

The number of points won on a single trial was calculated by:

$$\frac{100*number of spacebar presses}{b}$$

where b was the average number of times the spacebar was pressed during the 3 baseline button-pressing trials. This was to roughly equate the number of points each participant won per button-press. 400 points were required for one unit of each reward: ¼ cigarette, 30s music, ½ chunk of chocolate and 1 piece of paper. Malboro Gold cigarettes (tar 6 mg, nicotine 0.5 mg), Cadbury’s Dairy Milk chocolate, individually chosen music rated ≥75/100 in terms of ‘liking’ (Perkins and Karelitz 2013), and pieces (~2cm^2^) of lined paper were the real delivered rewards awarded after the task. Paper was included as a neutral commodity to demonstrate that participants of both groups were motivated by all of the reward available.

**Cigarette Purchase Task**

The instructions were as follows:

“Imagine that you could smoke RIGHT NOW AND FOR THE NEXT 3 HOURS. The following questions ask how many cigarettes you would consume if they cost various amounts of money. Assume the available cigarettes are your favourite brand. Assume that you have the same income/savings that you have now and NO ACCESS to any other cigarettes or nicotine products. In addition, assume that you cannot save or stockpile cigarettes for a later date after the 3 hours us up. Answer each question individually, i.e. the amount of cigarettes you would buy for price X should not affect the amount of cigarettes you would buy for price Y. remember it is asking you about how many cigarettes you would smoke RIGHT NOW AND FOR THE NEXT 3 HOURS.”

Participants were asked “How many cigarettes would you smoke if they were _____ each”. Prices included: £0 (free), 1p, 2p, 5p, 10p, 15p, 20p, 25p, 30p, 35p, 40p, 45p, 50p, 60p, 70p, 80p, 90p, £1, £2, £3, £4, £5, and were presented in that order.

The CPT produces a demand curve, reflecting the willingness to spend money on cigarettes at varying prices. The CPT generates five indices: (1) breakpoint, i.e. the price at which the number of cigarettes bought becomes zero; (2) intensity, i.e. the number of cigarettes bought at price £0; (3) O_max_, i.e. maximum expenditure; (4) P_max_, i.e. the price at which expenditure is maximum; and (5) elasticity, i.e. the sensitivity of cigarette purchase to increases in cost. The first four variables are calculated by directly observing participants’ behaviour on the task. Elasticity was calculated using the exponential demand equation ([Hursh and Silberberg 2008](#_ENREF_37)):

$${Log}_{10}Q={Log}_{10}Q_{0}+k(e^{-\alpha Q_{0}c}-1)$$

where Q=consumption at a given price, c; Q_0_=consumption when the price is zero; k=a constant across individuals set at 4.0 (http://www.ibrinc.org/index.html); c=the price of the commodity; and α=elasticity: the derived demand parameter reflecting a standardised rate of decline of consumption.

**Trait measures**

*Beck depression inventory (BDI-II) (*[*Beck et al. 1996*](#_ENREF_8)*)*

This scale of depression severity consists of 21 items that are rated for their frequency between 0 and 3 in the last week. Higher scores reflect greater depression.

*Cigarette dependence scale (CDS-5) (*[*Etter et al. 2003*](#_ENREF_24)*)*

This scale of cigarette dependence consists of 5 items. Higher scores reflect greater cigarette dependence.

*Drug history*

Participants were asked about: (1) lifetime use; (2) number of lifetime exposures; (3) if used in lifetime, how many days used per month now.

*DSM-5 Tobacco use disorder (*[*DSM-5 American Psychiatric Association 2013*](#_ENREF_21)*)*

Participants were asked questions about whether 11 symptoms were present over the past 12 months. 2-3 symptoms = mild tobacco use disorder; 4-5 symptoms = moderate tobacco use disorder; 6+ symptoms = severe tobacco use disorder.

*Fagerstrom test for nicotine dependence (FTND) (*[*Heatherton et al. 1991*](#_ENREF_35)*)*

This scale consists of six items that are rated between 0 and 3, with total scores ranging from 0 (low dependence) to 10 (high dependence).

*Frequency and general liking of rewards*

Participants were asked how many days per week, on average, they smoked a cigarette, listened to some music or ate some chocolate. They were also asked how much they liked, in general, smoking a cigarette, listening to their favourite music and eating Diary Milk chocolate (from -10 ‘extremely dislike’ to +10 ‘extremely like’).

*Temporal experiences of pleasure scale (TEPS)*

This scale consists of 18 items that are rated between 1 (very false for me) and 6 (very true for me). There are two subscales: anticipatory and consummatory pleasure. Higher scores reflect greater ability to experience pleasure.

**Other assessments**

*Spot-the-word (*[*Baddeley et al. 1993*](#_ENREF_5)*)*

This test, which correlates highly with premorbid verbal intelligence, consists of pairs of items, one a word and one a non-word; participants select the item they think is a real word.

*Carbon monoxide*

Expired carbon monoxide (CO) levels were determined with a Bedfont Micro Smokerlyzer (Bedfont Scientific, Harrietsham, UK).

**Statistical analyses**

*Demographics and self-report data*

T-tests and Mann Whitney U-tests were used to compare dependent and occasional smokers on demographic variables. The DEQ subscales were analysed using mixed-design ANOVAs with a between-subjects factor of group and within-subjects factors of drug (placebo, pramipexole) and time (post-drug, post-consumption). MPSS and TCQ-SF subscales were analysed in the same way but time had an extra level (pre-drug, post-drug, post-consumption).

*DReaM-Choice task*

Choices and button-pressing data from the DReaM-Choice task were analysed using mixed-design ANOVAs with a between-subjects factor of group and within-subjects factors of drug and reward (cigarette, music, chocolate and paper). Following Lawn et al., (2015), when one reward type was never chosen, a button-pressing score of 0 was assigned. Reaction time data were analysed in the same way but without paper included as a level in the reward factor, as it was chosen very infrequently. We also explored the potentially moderating effect depression had on behaviour using ANCOVA. BDI was entered as the covariate and a custom model was created in which there was a main effect of BDI and BDI interacted with each term. We also used a Bayes factor calculator (<http://pcl.missouri.edu/bayesfactor>) to evaluate evidence in favour of the null hypotheses that pramipexole did not affect motivation for cigarettes. We calculated Bayes factors within each group for paired samples t-tests for number of cigarette choices and average number of button-press for cigarettes.

Only the first liking rating of each reward was analysed so that satiation effects did not affect the data. These liking data were analysed using mixed effects models to deal with missing data points, as some participants won rewards and did not consume any of them, e.g. an occasional smoker winning half a cigarette but not smoking any of it. The mixed effects models had group, drug and reward (and their interactions) as fixed factors and the intercept allowed to vary randomly.

*Cigarette purchase task*

First, any participants who responded incompatibly were removed (i.e. stating they would purchase the same number of cigarettes for every price; stating they would buy more cigarettes for a greater price than a smaller price; or clearly answering erroneously, e.g. ‘100 cigarettes for 1p and 0 cigarettes for 2p’). Second, elasticity was calculated using the Hursh and Silberberg (2008) equation described in the supplementary materials using Graphpad Prism 6. Third, any data points for breakpoint, intensity, O_max_, P_max_, and elasticity that were outliers (defined as Z > 3.29) were replaced with the nearest non-outlying value + one unit at the second decimal (cite Mackillop 2012). Fourth, breakpoint, intensity, O_max_ and P_max_ were all log_10_ transformed to improve the normality of their distributions and their residuals’ distributions. Fifth, mixed-design ANOVAs with a between-subjects factor of group and a within-subjects factor of drug were then used to analyse the logs of breakpoint, intensity, O_max_ and P_max_, and also elasticity. We also explored the potentially moderating effect depression had on behaviour using ANCOVA. BDI was entered as the covariate and a custom model was created in which there a main effect of BDI and BDI interacted with each term.

We also used a Bayes factor calculator (<http://pcl.missouri.edu/bayesfactor>) to evaluate evidence in favour of the null hypotheses that pramipexole did not affect cigarette demand. We calculated Bayes factors within each group for paired samples t-tests for each CPT metric.

*Correlations*

Within each group separately, we investigated the correlations between number of cigarettes smoked/day, general liking of smoking one cigarette, cigarette dependence scale and: (1) the number of choices for cigarettes in the DReaM-Choice task (averaged across both sessions), (2) the average button-pressing for cigarettes in the DReaM-Choice task (averaged across both sessions) and (3) each of the five outcomes from the CPT (averaged across both sessions).

In order to examine the validity of the DReaM-Choice task, we correlated the number of choices and button-pressing for cigarettes with the five outcomes from the CPT for the whole sample. We also carried out correlations between the different demand metrics of the CPT.

The alpha level was set to 0.005 to account for multiple tests.

**Results**

*Drug effects questionnaire*

For ‘feel drug’, there were main effects of drug (F_1, 38_=6.477, p=0.015), with greater ratings on the pramipexole condition than the placebo condition, and time (F_1, 38_=4.669, p=0.037), with greater ratings at post-drug than post-consumption. For ‘like drug’, there was a drug by time interaction (F_1, 38_=4.376, p=0.043). Within the pramipexole condition, ‘like drug’ ratings decreased from post-drug to post-consumption (t_39_=2.920, p=0.024), however they did not change within the placebo condition. For ‘dislike drug’, there was a main effect of drug (F_1, 38_=7.684, p=0.009), with greater ratings on the pramipexole condition than the placebo condition. For ‘want more drug’, there was a main effect of drug (F_1, 38_=8.017, p=0.007), with greater ratings on the placebo condition than the pramipexole condition. There were no main effects or interactions for ‘high’ ratings.

*DReaM-Choice*

*Choices*

The dependent smokers chose cigarettes more than music (t_19=_6.463, p<0.001) and chocolate (t_19_=5.703, p<0.001), while the occasional smokers chose chocolate more than cigarettes (t_18_=4.616, p<0.001) and music (t_18_=4.189, p<0.001). Overall, all rewards were chosen more than paper (ps<0.001) and cigarettes and chocolate were chosen more than music (ps<0.006).

*Average number of button-presses*

All rewards were pressed for more than paper (ps<0.001). The dependent smokers pressed for all rewards similarly while the occasional smokers pressed for chocolate more than cigarettes (t_18_=3.707, p=0.004).

*Time taken to choose reward^^[[1]](#footnote-1)^^*

There was an interaction between group and reward (F_2, 64_=13.069, p<0.001) and a main effect of reward (F_2, 64_=3.349, p=0.041). The dependent smokers chose cigarettes faster than occasional smokers (t_32_=3.16, p=0.003). Within the dependent smokers, cigarettes were chosen faster than music (t_17_=5.707, p<0.001) and chocolate (t_17_=3.853, p=0.002); whereas within the occasional smokers, chocolate was chosen marginally faster than cigarettes (t_16_=2.501, p=0.053). There were no effects of pramipexole on the time taken to choose any reward. Including BDI as a covariate had no effects.

*Cigarette Purchase Task*

BDI was associated with greater breakpoint (F_1, 30_=3.354, p=0.077), a greater intensity (F_1, 30_=7.364, p=0.011) and a greater O_max_ (F_1, 30_=6.150, p=0.019). Furthermore, for intensity there was a group by BDI interaction (F_1, 30_=5.057, p=0.032); the main effect of BDI was only significant in the occasional smokers (F_1, 14_=8.374, p=0.012), but not the dependent smokers.

*Correlations*

In the dependent group, none of the correlations with DReaM-Choice task outcomes were significant. In the occasional group, however, there was a correlation between general liking of cigarettes and cigarette button-pressing (r_18_=0.741, p<0.001) and a marginal correlation between general liking of cigarettes and number of cigarette choices (r_17_=0.557, p=0.010) (with α set to 0.005). The correlations between general liking of cigarettes and cigarette BP and choices were significantly larger in the occasional group than the dependent group (ps≤0.02).

Behaviour on the DReaM-Choice task, as assessed by the number of choices for cigarettes and the average number of button-presses for cigarettes, was consistently associated with performance on the CPT, as shown by table *F* and demonstrated in figure *B* in supplementary materials.

**Further comments**

*Group differences*

Dependent smokers were willing to sacrifice alternative rewards in favour of cigarettes, as demonstrated by the greater number of choices for cigarettes than chocolate and music. Contrastingly, occasional smokers chose chocolate more than cigarettes. These data support previous work highlighting the association between nicotine dependence and the willingness to choose cigarettes over an alternative reward, chocolate (Hogarth and Chase 2011; 2012). Despite the *difference in the balance of cigarette and non-drug reward processing* between the groups, there was no evidence to suggest that there was a difference in the motivation for non-drug rewards between the groups. The greater motivation for cigarettes in the dependent group would necessarily drive the number of choices for other rewards, including chocolate, down. The absence of differences in button-pressing for music or chocolate between the groups means there is an absence of evidence for deficient non-drug reward motivation in the dependent relative to the occasional smokers.

*Task sensitivity*

Our findings could question the sensitivity of the tasks used to assess motivation for cigarettes, given we did not find any effects of pramipexole. However, the DReaM-Choice task is sensitive to both nicotine abstinence and nicotine dependence ([Lawn et al. 2015](#_ENREF_45)) and the CPT is sensitive to nicotine dependence ([MacKillop et al. 2008](#_ENREF_56)), depletion of L-tyrosine ([Hitsman et al. 2008](#_ENREF_36)) and the presentation of tobacco cues ([MacKillop et al. 2012](#_ENREF_55)), amongst other manipulations. Hence, it is unlikely that we missed an effect as a result of insensitive or invalid tasks.

*Correlation results*

Our correlation results are in favour of the dissociation between motivation and pleasure in nicotine dependence ([Berridge and Robinson 1998](#_ENREF_8); [Robinson and Berridge 2008](#_ENREF_59)). The occasional smokers’ button-pressing for cigarettes in the DReaM-Choice task was significantly correlated with their self-reported general liking of smoking a single cigarette. Whereas, the dependent smokers’ button-pressing was not correlated with their general liking. Furthermore, the correlations between this task behaviour and general liking of cigarette smoking were different between the groups. This implies that nicotine dependence may be associated with more habitual drug-seeking ([Everitt and Robbins 2005](#_ENREF_19)), in which there is a divergence between pleasure and choice behaviour.

*Depression*

It is noteworthy that higher depression was associated with greater demand for cigarettes in the CPT. Depression is related to smoking rates in epidemiological studies ([Fergusson et al. 2003](#_ENREF_26)) and anhedonia, a cardinal symptom of depression, predicts smoking cessation outcome ([Leventhal et al. 2014](#_ENREF_50); [Leventhal et al. 2008](#_ENREF_51)). This study demonstrates that the CPT has the sensitivity to detect the important relationship between depression level and motivation to smoke cigarettes. Importantly, inclusion of BDI as a covariate did not alter the effects of group in the DReaM-Choice task results, despite significant covariance between group and BDI.

*Table A Group means (SD) for demographic data for dependent and occasional smokers.*

|  | Dependent | Occasional |
| --- | --- | --- |
| Age | 24.35 (6.81) | 22.60 (3.79) |
| Gender (m/f) | 10/10 | 10/10 |
| Body mass index | 23.19 (3.24) | 22.86 (2.91) |
| Annual income (£) | 12,682 (8,210) | 13,111 (7,812) |
| Years in education | 15.83 (2.68) | 16.35 (1.87) |
| Spot-the-word | 49.00 (4.10) | 50.74 (3.55) |
| Cigarettes/day*** | 16.45 (5.80) | 0.54 (0.12) |
| Cigarettes/week*** | 115.15 (40.62) | 3.78 (0.83) |
| Age started smoking (years)* | 13.40 (2.44) | 15.03 (1.58) |
| FTND*** | 5.70 (1.03) | 0 |
| CDS-5*** | 18.53 (3.12) | 7.75 (2.51) |
| DSM-5*** | 6.20 (1.85) | 1.20 (1.00) |
| BDI* | 10.80 (7.50) | 6.26 (4.58) |
| TEPS anticipatory | 40.79 (6.55) | 40.35 (5.95) |
| TEPS consummatory | 38.42 (6.34) | 36.10 (5.81) |
| TEPS total | 79.53 (11.23) | 76.45 (9.65) |

**p<0.05, ***p<0.001. FTND Fagestrom test for nicotine dependence; CDS-5 Cigarette dependence scale; DSM-5 Diagnostic and statistical manual tobacco use disorder; BISBAS Behavioural activation/inhibition systems scale; TEPS Temporal experience of pleasure scale; BSSS brief sensation seeking scale; BIS Barratt impulsiveness scale*

*Table B Group means (SD) for the frequency and general liking of rewards for dependent and occasional smokers*

|  | Dependent | Occasional |
| --- | --- | --- |
| Like smoking one cigarette, in general (-10 to 10)*** | 8.21 (1.58) | 5.15 (2.39) |
| Like listening to one song of favourite music, in general (-10 to 10) | 8.26 (2.02) | 7.20 (2.35) |
| Like eating Diary Milk chocolate, in general (-10 to 10) | 1.84 (5.93) | 4.25 (2.73) |
| Days/week smoke at least one cigarette*** | 7.00 (0.00) | 2.73 (1.50) |
| Days/week listen to music | 6.47 (1.17) | 6.20 (1.54) |
| Days/week eat chocolate | 2.37 (1.61) | 2.70 (1.63) |

****p<0.001*

*Table C Group means (SD) for MPSS pre-drug, post-drug and post-consumption for placebo and pramipexole sessions for dependent and occasional smokers*

|  |  | Dependent | | Occasional | | Group X Drug X Time | Group X Drug | Group X Time | Drug X Time | Group | Drug | Time |
| --- | --- | --- | --- | --- | --- | --- | --- | --- | --- | --- | --- | --- |
|  |  | Placebo | Pramipexole | Placebo | Pramipexole | F_2, 76_ | F_1, 38_ | F_2, 76_ | F_2, 76_ | F_1, 38_ | F_1, 38_ | F_2, 76_ |
| MPSS depressed | Pre-drug | 1.50 (0.83) | 1.45 (0.69) | 1.40 (0.60) | 1.50 (0.76) | 0.207 | 0.053 | 1.484 | 0.798 | 0.832 | 0.000 | 8.376*** |
|  | Post-drug | 1.25 (0.55) | 1.30 (0.57) | 1.25 (0.55) | 1.35 (0.67) |  |  |  |  |  |  |  |
|  | Post-consumption | 1.20 (0.70) | 1.25 (0.55) | 1.25 (0.55) | 1.20 (0.41) |  |  |  |  |  |  |  |
| MPSS irritable | Pre-drug | 1.25 (0.55) | 1.30 (0.66) | 1.40 (0.68) | 1.75 (0.72) | 0.375 | 0.907 | 2.384 | 0.214 | 0.200 | 0.347 | 3.328* |
|  | Post-drug | 1.55 (0.76) | 1.65 (0.67) | 1.45 (0.69) | 1.50 (0.95) |  |  |  |  |  |  |  |
|  | Post-consumption | 1.35 (0.67) | 1.35 (0.59) | 1.55 (0.89) | 1.70 (1.03) |  |  |  |  |  |  |  |
| MPSS restless | Pre-drug | 1.90 (0.79) | 1.75 (0.85) | 1.90 (0.79) | 1.75 (0.72) | 0.600 | 0.770 | 2.035 | 0.308 | 0.377 | 0.159 | 3.469* |
|  | Post-drug | 1.85 (1.09) | 1.75 (0.91) | 1.75 (0.85) | 1.65 (1.09) |  |  |  |  |  |  |  |
|  | Post-consumption | 1.80 (1.01) | 1.85 (0.88) | 2.25 (1.07) | 2.10 (1.17) |  |  |  |  |  |  |  |
| MPSS hungry | Pre-drug | 1.95 (1.15) | 1.70 (0.73) | 2.15 (0.93) | 1.70 (0.92) | 0.054 | 0.139 | 0.389 | 1.079 | 0.006 | 1.468 | 36.516*** |
|  | Post-drug | 2.85 (1.09) | 2.65 (1.18) | 2.90 (1.21) | 2.55 (1.00) |  |  |  |  |  |  |  |
|  | Post-consumption | 2.95 (1.00) | 2.95 (1.15) | 2.85 (1.18) | 2.80 (1.44) |  |  |  |  |  |  |  |
| MPSS poor concentration | Pre-drug | 1.95 (0.94) | 1.80 (1.01) | 1.60 (0.82) | 1.70 (0.66) | 1.098 | 0.270 | 0.358 | 0.850 | 0.394 | 1.591 | 6.801** |
|  | Post-drug | 1.95 (0.89) | 2.30 (1.08) | 1.95 (0.94) | 2.10 (0.85) |  |  |  |  |  |  |  |
|  | Post-consumption | 1.95 (1.00) | 2.35 (0.92) | 2.10 (0.91) | 2.10 (0.79) |  |  |  |  |  |  |  |
| MPSS time spent with urges | Pre-drug | 2.35 (0.82) | 2.35 (0.75) | 0.40 (0.50) | 0.70 (0.66) | 1.375 | 0.033 | 0.220 | 0.867 | 100.103*** | 1.619 | 3.415* |
|  | Post-drug | 2.40 (1.27) | 2.65 (0.88) | 0.50 (0.69) | 0.55 (0.69) |  |  |  |  |  |  |  |
|  | Post-consumption | 2.60 (0.75) | 2.75 (1.02) | 0.70 (0.80) | 0.65 (0.67) |  |  |  |  |  |  |  |
| MPSS strength of urges to smoke | Pre-drug | 2.30 (0.73) | 2.40 (0.94) | 0.50 (0.69) | 0.90 (0.79) | 1.717 | 0.083 | 1.680 | 0.191 | 73.329*** | 5.756* | 0.288 |
|  | Post-drug | 2.30 (1.26) | 2.60 (0.88) | 0.45 (0.60) | 0.60 (0.75) |  |  |  |  |  |  |  |
|  | Post-consumption | 2.35 (0.99) | 2.65 (0.99) | 0.60 (0.68) | 0.60 (0.60) |  |  |  |  |  |  |  |

**p<0.05, **p<0.01, ***p<0.001. MPSS Mood and physical symptoms scale*

*Table D F tests, p values and partial eta squared values for (a) the main effect of drug and the (b) interaction between drug and group on CPT metrics. None of these reached significance (ps>0.05).*

*a) Drug*

| CPT metric | F [df_1_, df_2_] | p | η^2^_p_ |
| --- | --- | --- | --- |
| Breakpoint | 0.024 [1,32] | 0.878 | 0.001 |
| Intensity | 2.147 [1,32] | 0.153 | 0.063 |
| O_max_ | 0.278 [1,31] | 0.602 | 0.009 |
| P_max_ | 0.664 [1,31] | 0.421 | 0.021 |
| Elasticity | 2.674 [1,31] | 0.112 | 0.079 |

*b) Drug X Group*

| CPT metric | F [df_1_, df_2_] | p | η^2^_p_ |
| --- | --- | --- | --- |
| Breakpoint | 0.004 [1,32] | 0.949 | <0.001 |
| Intensity | 0.771 [1,32] | 0.386 | 0.024 |
| O_max_ | 1.585 [1,31] | 0.217 | 0.049 |
| P_max_ | 3.146 [1,31] | 0.086 | 0.092 |
| Elasticity | 3.766 [1,31] | 0.061 | 0.108 |

*Table E Correlations between performance on the DReaM-Choice, as assessed by the number of cigarette choices and the average number of button-presses for cigarettes, and performance on the CPT, as assessed by the log_10_ scores of breakpoint, intensity, O_max_ and P_max_, and elasticity (collapsed across drug condition and group).*

****p<0.001; **p<0.01; *p<0.05*

*Table F Correlations between demand indices (collapsed across drug condition and group). Breakpoint, Intensity, O_max_ and P_max_ were log_10_ transformed.*

|  | Breakpoint | Intensity | O_max_ | P_max_ | Elasticity |
| --- | --- | --- | --- | --- | --- |
| Number of cigarette choices | 0.737*** | 0.561** | 0.701*** | 0.696*** | -0.615*** |
| Average number of button-presses for cigarettes | 0.452** | 0.454** | 0.384* | 0.363* | -0.436* |

****p<0.001; **p<0.01; *p<0.05*

| Variable | 1 | 2 | 3 | 4 |
| --- | --- | --- | --- | --- |
| 1 Breakpoint | - | - | - | - |
| 2 Intensity | 0.674*** | - | - | - |
| 3 *O_max_* | 0.907*** | 0.745*** | - | - |
| 4 *P_max_* | 0.936*** | 0.515** | 0.873*** | - |
| 5 Elasticity | -0.584** | -0.259 | -0.616*** | -0.582*** |

*Figure A Group means for liking of (-10 ‘extremely dislike’ to +10 ‘extremely like’) the first consumed unit of cigarette, music and chocolate reward, in the DReaM-Choice, for dependent and occasional smokers on the placebo and pramipexole sessions. Error bars show standard error.*

*Figure B The correlation between the average number of button-presses for cigarettes in the DReaM-Choice task and the log_10_(*O_max_*) scores in the CPT. Circles represent dependent smokers and triangles represent occasional smokers.*

1. Six participants were excluded due to never choosing one of the reward types. [↑](#footnote-ref-1)
